# Supplementary material for: Missense variants in TUBA4A cause myo-tubulinopathies
Source: medRxiv. 2025 Jun 28:2025.06.26.25330266. Preprint. [Version 1] doi: 10.1101/2025.06.26.25330266 (PMC12262762; doi:10.1101/2025.06.26.25330266)
Supplement: 1 [file NIHPP2025.06.26.25330266V1-supplement-1.pdf]

Supplementary Table: Assessment of variants in TUBA4A observed in our cohort. AL=Acceptor Loss, AG=Acceptor Gain, AR = Autosomal recessive, AD = Autosomal dominant, n=number of confirmed affected individuals, N=number of families, NA=Not assessed.

		Population fequency	Raw Classification		In silico evidence								*In vitro* evidence		Clinical evidence			Variants in other alphaTubulins	Revised Classification	
**TUBA4A variants NM_006000.3**	**hg38 coordinates**	**gnomAD4.1.0**	**Assertion**	**Rules**	**AlphaMissense**	**CADD**	**REVEL**	**spliceAI**	**Free Energy**	**Polar contacts**	**Steric hindrance**	**Aggrescan 4D of residue**	**Morphology in COS1**	**Patient IDs**	**Phenotype**	**type/segregation**	**n[N]**		**Assertion**	**add Rules**
de novo																				
c.33G>C(p.Gln11His)	chr2-219252201-C-G	0	VUS	PP3_Moderate,PM2_Supporting,PP2	0.9967	24.4	0.808	0	Positive	addition	Yes	"-0.742" (Soluble residue)	NA	Pat.24	Multisystem proteinopathy	heterozygous	1[1]		LP	PS2_strong
c.679C>T(p.Leu227Phe)*	chr2-219251020-G-A	0	VUS	PP3_Moderate,PM2_Supporting,PP2	0.9921	26.7	0.755	0	High Positive	no change	Yes	0.5505 (aggregation prone residue)	abnormal-mild	Pat.7	Myopathy	heterozygous	1[1]	*TUBA1A*:(p.(Leu227Gln)) is a VUS	P	PS2_strong, PS3_strong, PP1_supporting
c.761A>G(p.Glu254Gly)	chr2-219250938-T-C	0	LP	PP3_Strong,PM2_Supporting,PP2	0.989	29.3	0.929	AL=0.02	Positive	loss	No	"-0.5888" (Soluble residue)	normal-mild	Pat.10	Myopathy	heterozygous	1[1]		P	PS2_strong, PS3_strong
c.760G>A(p.Glu254Lys)	chr2-219250939-C-T	0	VUS	PP3_Moderate,PM2_Supporting,PP2	0.999	26.7	0.846	0	none	loss	No	"-1.4057" (Soluble residue)	mild	Pat.8	Myopathy	heterozygous	1[1]		P	PS2_strong, PS3_strong
c.1049G>T(p.Gly350Val)	chr2-219250650-C-A	0	LP	PP3_Strong,PM2_Supporting,PP2	0.999	26	0.893	0	Positive	no change	Yes	"-0.5039" (soluble residue)	NA	Pat.25	Myopathy	heterozygous	1[1]	*TUBA1A*:(p.(Gly350Val)) is pathogenic	P	PS2_strong
Sporadic																				
c.536C>T(p.Thr179Ile)*	chr2-219251163-G-A	0	VUS	PP3_Moderate,PM2_Supporting,PP2	0.9657	25.3	0.731	AL=0.06/AG=0.02	Negative	none	No	2.1503 (aggregation prone residue)	abnormal-mild	Pat.5	Myopathy	heterozygous	1[1]		P	PS3_strong, PP1_supporting
c.679C>T(p.Leu227Phe)*	chr2-219251020-G-A	0	VUS	PP3_Moderate,PM2_Supporting,PP2	0.9921	26.7	0.755	0	High Positive	no change	Yes	0.5505 (aggregation prone residue)	abnormal-mild	Pat.6	Myopathy	heterozygous	1[1]	*TUBA1A*:(p.(Leu227Gln)) is a VUS	P	PS2_strong, PS3_strong, PP1_supporting
c.761A>C(p.Glu254Ala)	chr2-219250938-T-G	0	LP	PP3_Strong,PM2_Supporting,PP2	0.9895	27.4	0.941	0	none	loss	No	"-0.6042" (Soluble residue)	normal-mild	Pat.11	Myopathy	heterozygous	1[1]		P	PS3_strong
c.760G>C(p.Glu254Gln)	chr2-219250939-C-G	0	VUS	PP3_Moderate,PM2_Supporting,PP2	0.9905	23.5	0.729	0	none	loss	No	"-1.612" (Soluble residue)	normal-mild	Pat.9	Myopathy	heterozygous	1[1]		LP	PS3_strong
c.849C>G(p.His283Gln)	chr2-219250850-G-C	0	LP	PP3_Strong,PM2_Supporting,PP2	0.9923	17.92	0.735	0	Negative	addition	No	"-1.662" (Soluble residue)	NA	Pat.12	Myopathy	heterozygous	1[1]	*TUBA1A*: (p.(His283Arg)) is Liklely pathogenic	LP	none
c.850G>A(p.Glu284Lys)*	chr2-219250849-C-T	0	VUS	PP3_Moderate,PM2_Supporting,PP2	0.9894	27.1	0.824	0	Negative	none	No	"-2.6099" (Soluble residue)	NA	Pat.13 and Pat.14	Myopathy	heterozygous	2[2]	*TUBA8*:(p.(Glu284Lys)) is VUS	VUS	PP1_supporting
Autosomal Recessive																				
c.34G>A(p.Ala12Thr)	chr2-219252200-C-T	0	VUS	PP3_Moderate,PM2_Supporting,PP2	0.9451	26.8	0.832	0	Positive	addition	Yes	"-0.4499" (Soluble residue)	NA	Pat.26	Multisystem proteinopathy	homozygous/AR	1[1]		VUS	none
c.722C>T(p.Ser241Phe)	chr2-219250977-G-A	0	VUS	PP3_Moderate,PM2_Supporting,PP2	0.9989	28.3	0.795	0	High Negative	loss	Yes	0 (Soluble residue)	abnormal	Pat.21	Myopathy	homozygous/AR	1[1]		P	PS3_strong, change PP3_strong
c.1061G>A(p.Gly354Asp)	chr2-219250638-C-T	0	LP	PP3_Strong,PM2_Supporting,PP2	0.9996	26.1	0.901	0	Negative	addition	Yes	0 (Soluble residue)	abnormal	Pat.22 and Pat.23	Myopathy	homozygous/AR	2[1]		P	PS3_strong
Autosomal Dominant																				
c.536C>T(p.Thr179Ile)*	chr2-219251163-G-A	0	VUS	PP3_Moderate,PM2_Supporting,PP2	0.9657	25.3	0.731	AL=0.06/AG=0.02	Negative	none	No	2.1503 (aggregation prone residue)	abnormal-mild	Pat.2	Myopathy	heterozygous/AD	4[1]		P	PS3_strong, PP1_supporting
c.850G>A(p.Glu284Lys)*	chr2-219250849-C-T	0	VUS	PP3_Moderate,PM2_Supporting,PP2	0.9894	27.1	0.824	0	Negative	none	No	"-2.6099" (Soluble residue)	NA	Pat.15, Pat16A, Pat.16B, Pat.17 and Pat.18	Myopathy	heterozygous/AD	11[3]	*TUBA8*:(p.(Glu284Lys)) is VUS	VUS	PP1_supporting

* indicates recurring variants
